# Overtime Work and Prevalence of Diabetes in Japanese Employees: Japan Epidemiology Collaboration on Occupational Health Study

**DOI:** 10.1371/journal.pone.0095732

**Published:** 2014-05-01

**Authors:** Keisuke Kuwahara, Teppei Imai, Akiko Nishihara, Tohru Nakagawa, Shuichiro Yamamoto, Toru Honda, Toshiaki Miyamoto, Takeshi Kochi, Masafumi Eguchi, Akihiko Uehara, Reiko Kuroda, Daisuke Omoto, Kayo Kurotani, Ngoc Minh Pham, Akiko Nanri, Isamu Kabe, Tetsuya Mizoue, Naoki Kunugita, Seitaro Dohi, Japan Epidemiology Collaboration on Occupational Health Study Group

**Affiliations:** 1 Department of Epidemiology and Prevention, Center for Clinical Sciences, National Center for Global Health and Medicine, Tokyo, Japan; 2 Azbil corporation, Tokyo, Japan; 3 Hitachi, Ltd., Ibaraki, Japan; 4 Nippon Steel & Sumitomo Metal Corporation Kimitsu Works, Chiba, Japan; 5 Furukawa Electric Corporation, Tokyo, Japan; 6 YAMAHA CORPORATION, Shizuoka, Japan; 7 The University of Tokyo, Tokyo, Japan; 8 Mitsubishi Heavy Industries, Ltd., Aichi, Japan; 9 Department of Epidemiology, Faculty of Public Health, Thai Nguyen University of Medicine and Pharmacy, Thai Nguyen Province, Vietnam; 10 National Institute of Public Health, Saitama, Japan; 11 Mitsui Chemicals, Inc., Tokyo, Japan; University of North Carolina at Chapel Hill, United States of America

## Abstract

**Objective:**

Epidemiologic evidence on long working hour and diabetes has been conflicting. We examined the association between overtime work and prevalence of diabetes among Japanese workers.

**Methods:**

The subjects were 40,861 employees (35,170 men and 5,691 women), aged 16 to 83 years, of 4 companies in Japan. Hours of overtime were assessed using self-reported questionnaires. Diabetes was defined as a fasting plasma glucose ≥126 mg/dl (7.0 mmol/l), hemoglobin A1c ≥6.5% (48 mmol/mol), or current use of anti-diabetic drug. Multiple logistic regression analysis was used to calculate odds ratio of diabetes for each category of overtime.

**Results:**

After adjustment for age, sex, company, smoking, and BMI, there was a suggestion of U-shaped relationship between overtime work and prevalence of diabetes (P for quadratic trend = 0.07). Compared with those who worked <45 hours of overtime per month, the adjusted odds ratios (95% confidence interval) of diabetes were 0.86 (0.77–0.94), 0.69 (0.53–0.89), and 1.03 (0.72–1.46) for those who worked 45–79, 80–99, and ≥100 hours of overtime per month, respectively. In one company (n = 33,807), where other potential confounders including shift work, job position, type of department, alcohol consumption, sleep duration, leisure time physical activity, and family history of diabetes was additionally adjusted for, similar result was obtained (P for quadratic trend = 0.05).

**Conclusions:**

Long hours of overtime work may not be associated with increased prevalence of diabetes among Japanese workers.

## Introduction

Working long hours has been paid much attention in relation to cardiovascular risk or *karoshi*, death due to overwork [1], [2], possibly caused by short sleep duration, physical inactivity, and prolonged exposure to psychological stress at work [3], [4]. Because such deterioration of lifestyle has been linked to diabetes [5], [6], [7], working long hours may also increase the risk of diabetes. Given working long hours is still common in many countries including Japan [8] and that the prevalence of diabetes has been increasing [9], causing a significant health and economic burden [10], [11], [12], it is important to uncover whether working long hours increases risk of diabetes.

Several studies have addressed the relation of working hours to the risk of diabetes, but their results are inconsistent. A Japanese study [13] and a US study [14] reported an increased risk of diabetes with increasing working hours. In contrast, other Japanese studies found no association [15] or even decreased risk of diabetes [16] with increasing working hours. Some methodological issues in these studies need to be addressed. One is that no previous study has examined the association with extremely long overtime (for instance, ≥100 hours per month) in relation to diabetes. Another is that no study used hemoglobin A1c (HbA1c), a diagnostic test for diabetes recently adopted by the International Expert Committee [17]. Furthermore, sample size in half of previous studies was not large (n = 1,000 to 2,000) [13], [16], which may limit their findings.

Here, we cross-sectionally investigated associations between overtime work and prevalence of diabetes among a Japanese working population using a large-scale multiple companies-based data using fasting glucose and HbA1c criterion. We hypothesized that long working hours would be associated with increased prevalence of diabetes. Nonetheless, preventive measures for overtime work related health problem were introduced by the Japanese government in 2002–2005 [18], after or during the all the Japanese studies were conducted. Such political actions may modify the relation of working hours with diabetes.

## Methods

### Study Procedure

Japan Epidemiology Collaboration on Occupational Health (J-ECOH) Study is an ongoing, large-scale study among workers in Japan. According to standard procedure of the study, researchers obtained several types of worker health data including those of periodic health checkup (2008 and thereafter), cardiovascular event (myocardial infarction and stroke), death from all causes, and long-term sick leave (1 month or longer) from participating companies. Additional researches including case-control study on cardiovascular event and nutritional survey were performed in selected companies.

In Japan, employees are obliged to undergo general health examination at least once a year under the health and safety law. Of the 11 participating companies of the J-ECOH Study, 9 provided data on periodic health checkup that was performed during the period between 2008 and 2011, which was then combined to create an analytic database. Of these, data with the earliest date of examination (mostly in 2008) were selected for the present cross-sectional analysis; however, if 2008 dataset for a company contained a much greater number of missing information or much fewer subjects than other datasets, 2009 or 2010 dataset were used instead, giving a total sample size of 80,469 (67,472 men and 12,997 women aged 15–84 years) for cross-sectional analysis.

### Ethics Statement

Prior to the collection of data, the conduct of the J-ECOH Study was announced in each company by using posters that explained the purpose and procedure of the study. Participants did not provide their verbal or written informed consent to join the study but were allowed to refuse their participation. This procedure conforms to the Japanese Ethical Guidelines for Epidemiological Research [19], where informed consent is not necessarily required for observational studies using existing data. The study protocol was approved by the Ethics Committee of the National Center for Global Health and Medicine, Japan. Most participating companies provided data in either anonymized or de-identified form, but a few other companies provided data including identifiable information, which was removed from analytic database. The data are hosted in the National Center for Global Health and Medicine. Currently, the data cannot be widely shared because the research group has not obtained permission from participating companies to provide the data on request. However, the data can be requested by other researchers for the purpose of academic, non-commercial research; inquiries and applications can be made to Department of Epidemiology and Prevention, Center for Clinical Sciences, National Center for Global Health and Medicine, Tokyo, Japan.

### Subjects

We extracted checkup data for 64,434 individuals in 4 companies where overtime work data were available from the original database. Of these, we excluded data for 1,809 participants who reported a history of cardiovascular disease (n = 490), cerebrovascular disease (n = 182), or psychiatric disease (n = 1,160). After this manipulation, we further excluded 21,764 subjects who had missing data on overtime work (n = 7,604), HbA1c (n = 12,768), blood glucose (n = 11,871), current use of anti-diabetic drug (n = 1,261), body mass index (BMI) (n = 138), smoking status (n = 6,032), and fasting condition (n = 1,449); and who received health checkup in non-fasting condition (n = 4,592). Some participants met more than one of the exclusion criteria. Finally, 40,861 participants (35,170 men and 5,691 women) remained for analysis.

### General Health Examination

Body height and body weight were measured according to a standard protocol in each company. BMI was calculated as weight in kilograms divided by squared height in meters. History of disease and health-related lifestyle were ascertained using a questionnaire, the content of which differs considerably among participating companies. Biochemical measurements included plasma glucose and HbA1c. HbA1c was measured according to a method used by the Japan Diabetes Society, thus we converted it to the National Glycohemoglobin Standardization Program (NGSP) equivalent value (%) using the formula: HbA1c (%)  = 1.02×HbA1c (Japan Diabetes Society) (%) +0.25% [20].

### Overtime Work Hours

In two companies, employees self-reported overtime work hours at health checkup (<45, 45 to <60, 60 to <80, 80 to <100, or ≥100 hours per month in the last 2 to 3 months; <45, 45 to <80, 80 to <100, or ≥100 hours per month, respectively). In another company, employees self-reported average total working hours per day at the timing of health checkup and monthly overtime were calculated using the following formula: (daily hours worked – 8 h) ×20 days. In the remaining company, employees were asked to self-report their overtime work hours in the last month with 11 response options (from “0 to 10” to “>100 hours” per month) at annual survey, not health checkup, in September. We classified these data on overtime work hours into 4 categories using cutoff point of 45, 80, and 100 hours per month for statistical analysis. In one company, 41–50 hours and 71–80 hours of overtime was categorized into 45–79 hours, and >100 hours into ≥100 hours for statistical analysis.

### Diagnosis of Diabetes

Diabetes was diagnosed according to the American Diabetes Association criteria [21] as a fasting plasma glucose level of ≥126 mg/dl (≥7.0 mmol/L), HbA1c of ≥6.5% (≥48 mmol/mol), or the current use of anti-diabetic drug.

### Other Variables

Smoking status (never, past, or current) and, if current smoker, number of cigarettes smoked per day were asked at the time of health check-up.

Detailed information on job, lifestyle, and family history of disease was available in one of these companies and used for adjustment as a sensitivity analysis. The information on shift work, job position, type of department, alcohol consumption, sleep duration, physical activity, and family history of diabetes was assessed using a questionnaire at the time of health check-up. Shift work was categorized as shift worker (rotating or night shift) or non-shift worker. Job position was categorized as high position (department chief, department director, or more) or low position (others). We classified 12 departments into two categories; one was termed “field work” for 4 departments and the rest was termed “non-field work” for 8 departments. Averaged daily ethanol consumption from alcohol beverage was calculated as drinking frequency multiplied by ethanol consumption per drinking day. Average sleep duration were assessed with 4 options (<5 hours, 5–<6 hours, 6–<7 hours, or ≥7 hours). Total weekly minutes of leisure time physical activity were calculated as frequency of physical activity or sports activity multiplied by duration of the activity.

### Statistical Analysis

The difference of age, sex, smoking, and BMI between those who were included in the present analysis and those who were excluded was tested by using t-test for continuous variables and χ^2^ test for categorical variables. Means (standard error) and percentages across overtime category were presented for continuous and categorical variables with adjustment for age and sex, respectively. Trend association was assessed using linear regression analysis for continuous variable and using logistic regression for categorical variables by assigning 23, 62, 90, and 100 to categories of overtime work hours, respectively.

Multiple logistic regression analysis was performed to calculate odds ratio and its 95% confidence interval of having diabetes across categories of overtime work hours. Trend association was assessed by assigning 23, 62, 90, and 100 to each category of overtime. To test quadratic trend, we used Stata contrast command after running multiple logistic regression. Model 1 was adjusted for age (continuous, year), sex, and company (4 companies). Model 2 was additionally adjusted for smoking (never, past, or current) and model 3 for BMI (continuous, kg/m^2^).

In one company (n = 33,807) from which detailed information on work and lifestyle was obtained, we additionally adjusted for other potential confounders, including alcohol use (non-drinker, drinker consuming >0 to <23 g, 23 to <46 g, or ≥46 g of ethanol per day), family history of diabetes (yes or no), shift work (yes or no), department (field work or non-field work), and job position (high or low) in model 2. We further adjusted for sleep duration (<6 hours, 6 to <7 hours, or ≥7 hours per day) in model 3 and physical activity (<150 min or ≥150 min per week) in model 4. We examined the effect modification by shift work (yes or no), type of department (field work or non-field work), smoking habits (non-smoker or smoker), alcohol use (<23 g or ≥23 g of ethanol/day), physical activity (<150 min or ≥150 min per week), and sleep duration (<6 hours or ≥6 hours) on the association between overtime work and diabetes using likelihood ratio test comparing models with and without interaction terms in the fully adjusted model treating overtime work as a categorical variable. We repeated the above analyses after exclusion of subjects under medication for diabetes to minimize the possibility of reverse causality. Two-sided P values of less than 0.05 were considered as statistically significant. All analyses were performed using Stata version 12.1 (StataCorp, College Station, Texas, USA).

## Results

Compared with those who were included in the present study (n = 40,861), those who were excluded (n = 23,573) were younger (34.6 years vs. 45.4 years), tended to be female (22.4% vs. 13.9%) and non-smoker (crude smoking rates: 35.9% vs. 40.3%), and had a lower BMI (22.9 kg/m^2^ vs. 23.5 kg/m^2^).

Table 1 shows the characteristics of participants according to overtime working hours per month with adjustment for age and sex. Subjects with long overtime were younger and more likely to be male, non-smokers, and physically inactive in leisure time, slept shorter hours, and had family history of diabetes compared with those with short overtime. As regard work related variables, subjects who worked long overtime tended to be a non-shift worker, in a high job position, and in non-field work-related department than those who worked short overtime.

**Table 1:** Subject characteristics according to overtime work hours.

	Overtime work (hours per month)				
Characteristic	<45	45–79	80–99	≥100	*P* for trend*
No. of subjects	29,308	9,648	1,369	536	
Male sex, %	81.5	97.6	98.2	98.6	<0.001
Age, year,	46.4 (0.1)	42.8 (0.1)	42.5 (0.3)	42.8 (0.4)	<0.001
Body-mass index, kg/m^2^	23.5 (0.02)	23.4 (0.03)	23.6 (0.09)	23.6 (0.14)	0.74
Body-mass index ≥25 kg/m^2^, %	28.0	26.8	28.0	27.1	0.1
Current smoker, %	40.2	37.2	31.4	32.7	<0.001
Alcohol user (≥23 g of ethanol/day), %†	23.6	25.1	22.7	20.1	0.38
Sleeping <6 hours per day, %†	45.5	63.1	80.5	86.3	<0.001
Leisure time physical activity, %† ^,^ ‡	15.2	11.9	8.1	6.5	<0.001
Family history of diabetes, %†	16.0	16.6	17.9	17.5	0.025
Shift worker, %†	18.0	13.5	8.3	10.1	<0.001
Field-work department, %†	47.8	42.4	31.0	30.3	<0.001
High job position, %†	10.0	22.2	34.8	36.3	<0.001

Data are adjusted for age and sex, and presented as mean ± standard error unless otherwise specified.

*P for trend was obtained from linear regression for continuous variables, or from logistic regression for categorical variables. by assigning 23, 62, 90, and 100 to categories of overtime work.

† n = 33,807 in one company.

‡ Defined as ≥150 min per week.

Table 2 shows the associations between overtime work and prevalence of diabetes. In the age-, sex-, and company-adjusted model, there was a U-shaped manner (P for quadratic trend = 0.038). The adjusted odds ratios (95% confidence intervals) were 1.00 (reference), 0.84 (0.76, 0.93), 0.67 (0.52, 0.87), and 1.03 (0.74, 1.45) for those who worked <45, 45 to <80, 80 to <100, and ≥100 hours of overtime, respectively. After further adjustment for smoking and BMI, the U-shaped relationship remained but became marginally significant (P for quadratic trend = 0.07). In one company (n = 33,807), additional adjustment for shift work, job position, type of department, family history of diabetes, alcohol use, sleep duration, and leisure time physical activity did not appreciably alter the associations in model 4 (P for quadratic trend = 0.05). The corresponding odds ratios were 1.00 (reference), 0.88 (0.79, 0.98), 0.67 (0.49, 0.90), and 1.12 (0.77, 1.63) for those who worked <45, 45 to <80, 80 to <100, and ≥100 hours of overtime, respectively. After an exclusion of 1,609 subjects who were under treatment for diabetes, results were not materially changed (data not shown).

**Table 2:** Odds ratio (OR) and 95% confidence interval of diabetes* according to overtime work hours.

	Overtime work (hours per month)				
	<45	45–79	80–99	≥100	*P* for quadratic trend†
4 companies					
No. of subjects	29,308	9,648	1,369	536	
No. of cases (%)	2418 (8.3)	583 (6.0)	64 (4.7)	38 (7.1)	
Model 1‡					
Multivariable-adjusted OR	1.00	0.84	0.67	1.03	0.038
(95% confidence interval)	(Ref)	(0.76, 0.93)	(0.52, 0.87)	(0.74, 1.45)	
Model 2§					
Multivariable-adjusted OR	1.00	0.85	0.69	1.05	0.037
(95% confidence interval)	(Ref)	(0.77, 0.94)	(0.53, 0.89)	(0.75, 1.48)	
Model 3||					
Multivariable-adjusted OR	1.00	0.86	0.69	1.03	0.07
(95% confidence interval)	(Ref)	(0.77, 0.95)	(0.53, 0.89)	(0.72, 1.46)	
1 company					
No. of subjects	23,094	9,116	1,136	461	
No. of cases (%)	1,927 (8.3)	554 (6.1)	51 (4.5)	36 (7.8)	
Model 1¶					
Multivariable-adjusted OR	1.00	0.85	0.66	1.16	0.011
(95% confidence interval)	(Ref)	(0.77, 0.94)	(0.49, 0.87)	(0.82, 1. 64)	
Model 2**					
Multivariable-adjusted OR	1.00	0.89	0.70	1.17	0.045
(95% confidence interval)	(Ref)	(0.80, 1.00)	(0.52, 0.94)	(0.81, 1.70)	
Model 3††					
Multivariable-adjusted OR	1.00	0.87	0.66	1.11	0.05
(95% confidence interval)	(Ref)	(0.78, 0.97)	(0.49, 0.90)	(0.76, 1.61)	
Model 4‡‡					
Multivariable-adjusted OR	1.00	0.88	0.67	1.12	0.05
(95% confidence interval)	(Ref)	(0.79, 0.98)	(0.49, 0.90)	(0.77, 1.63)	

Abbreviations: OR, odds ratio; Ref, reference.

*Defined as fasting glucose ≥126 mg/dL (7.0 mmol/l), HbA1c ≥6.5% (48 mmol/mol), or current use of anti-diabetic drug.

† *P* for quadratic trend obtained from multiple logistic regression analysis by assigning 23, 62, 90, and 100 to categories of overtime work.

‡ Model 1 adjusted for age (continuous), sex, and company in 4 companies (n = 40,861).

§ Model 2 adjusted for factors in model 1 and smoking status (never, past, or current) in 4 companies (n = 40,861).

|| Model 3 adjusted for factors in model 2 and body mass index (kg/m^2^, continuous) in 4 companies (n = 40,861).

¶ Model 1 adjusted for age (continuous) and sex in 1 company (n = 33,807).

**Model 2 adjusted for factors in model 1 plus smoking status (never, past, or current), body mass index (kg/m^2^, continuous), alcohol use (non-drinker, drinker consuming >0 to <23 g, 23 to <46 g, or ≥46 g of ethanol per day), family history of diabetes (yes or no), shift work (yes or no), department (field work or non-field work), and job position (high or low) in 1 company (n = 33,807).

†† Model 3 adjusted for factors in model 2 and sleep duration (<6 hours, 6 to <7 hours, or ≥7 hours per day) in 1 company (n = 33,807).

‡‡ Model 4 adjusted for factors in model 3 and leisure time physical activity (<150 min or ≥150 min per week) in 1 company (n = 33,807).

Table 3 shows odds ratio of diabetes across overtime work by risk factors for diabetes. There was no significant effect modification by age, sex, overweight, shift work, type of department, smoking status, alcohol consumption, sleep duration, and physical activity (all P for interaction >0.05).

**Table 3:** Odds ratio with 95% confidence interval of diabetes according to overtime work hours stratified by participant characteristics.

		Overtime work (hours per month)						
	No. of subjects	<45	45–79	80–99		≥100		*P* for quadratic trend*
4 companies								
Age		P for interaction = 0.98						
<40 years†	11835	1.00 (Ref)	0.88 (0.66, 1.17)	0.76 (0.40, 1.44)		0.95 (0.37, 2.44)		0.69
≥40 years†	29026	1.00 (Ref)	0.85 (0.76, 0.95)	0.67 (0.50, 0.89)		1.03 (0.70, 1.49)		0.07
Sex		P for interaction = 0.30						
Men†	35170	1.00 (Ref)	0.86 (0.77, 0.95)	0.70 (0.54, 0.91)		1.00 (0.70, 1.43)		0.09
Women†	5616‡	1.00 (Ref)	1.04 (0.47, 2.30)	-		5.21 (0.58, 47.24)		-
Smoking status		P for interaction = 0.36						
Non-smoker†	24399	1.00 (Ref)	0.78 (0.68, 0.90)	0.71 (0.51, 1.00)		0.96 (0.60, 1.55)		0.11
Current smoker†	16462	1.00 (Ref)	0.94 (0.81, 1.09)	0.64 (0.42, 0.97)		1.10 (0.66, 1.84)		0.33
BMI (kg/m^2^)		P for interaction = 0.32						
<25†	29437	1.00 (Ref)	0.84 (0.73, 0.97)	0.77 (0.53, 1.12)		1.03 (0.62, 1.73)		0.19
≥25†	11424	1.00 (Ref)	0.90 (0.78, 1.04)	0.65 (0.45, 0.94)		1.08 (0.67, 1.74)		0.22
1 company								
Shift work		P for interaction = 0.24						
Non-shift worker§	27718	1.00 (Ref)	0.86 (0.76, 0.97)	0.59 (0.42, 0.83)		1.16 (0.77, 1.73)		0.026
Shift worker§	6089	1.00 (Ref)	0.97 (0.75, 1.26)	1.32 (0.65, 2.67)		0.91 (0.32, 2.57)		0.86
Type of department		P for interaction = 0.93						
Field work§	15464	1.00 (Ref)	0.91 (0.78, 1.06)	0.68 (0.42, 1.12)		1.02 (0.55, 1.92)		0.45
Non-field work§	18343	1.00 (Ref)	0.83 (0.71, 0.97)	0.65 (0.44, 0.95)		1.13 (0.71, 1.80)		0.05
Alcohol use		P for interaction = 0.11						
<23 g of ethanol/day§	24674	1.00 (Ref)	0.93 (0.82, 1.06)	0.62 (0.43, 0.88)		1.24 (0.82, 1.88)		0.07
≥23 g of ethanol/day§	9133	1.00 (Ref)	0.73 (0.60, 0.90)	0.79 (0.46, 1.37)		0.73 (0.30, 1.77)		0.61
Sleep duration		P for interaction = 0.22						
<6 hours§	17592	1.00 (Ref)	0.87 (0.75, 1.00)	0.57 (0.39, 0.81)		1.12 (0.74, 1.69)		0.049
≥6 hours§	16215	1.00 (Ref)	0.88 (0.74, 1.04)	1.16 (0.66, 2.01)		1.12 (0.42, 2.99)		0.56
Physical activity		P for interaction = 0.06						
<150 min per week§	28963	1.00 (Ref)	0.89 (0.79, 1.00)		0.72 (0.53, 0.98)		1.16 (0.79, 1.70)	0.06
≥150 min per week§	4844	1.00 (Ref)	0.80 (0.60, 1.07)		0.13 (0.02, 0.94)		0.51 (0.06, 3.96)	0.95

Abbreviations: BMI, body mass index; Ref, reference.

*P for trend obtained from multiple logistic regression analysis by assigning 23, 62, 90, and 100 to categories of overtime work.

† Adjusted for age (continuous), sex, company, smoking status (never, past, or current), and BMI (kg/m^2^, continuous) in 4 companies (n = 41,081).

‡ 48 women in 1 company were excluded in this analysis due to no diabetic patients.

§ Adjusted for age (continuous), sex, company, smoking status (never, past, or current), BMI (kg/m^2^, continuous), alcohol use (non-drinker, drinker consuming >0 to <23 g, 23 to <46 g, or ≥46 g of ethanol per day), sleep duration (<6 hours, 6 to <7 hours, or ≥7 hours per day), physical activity (<150 min or ≥150 min per week), family history of diabetes (yes or no), shift work (yes or no), department (field work or non-field work), and job position (high or low) in 1 company (n = 33,807).

## Discussion

In the present study among Japanese workers, overtime work was associated with diabetes in a U-shaped manner, with the lowest odds ratio of diabetes being observed in persons with 80 to 99 hours of overtime per month. This study not only provides evidence on overtime and diabetes using a large dataset but also extends knowledge to extremely long overtime (100 hours or more per month).

We found a decreasing trend of diabetes prevalence with increasing hours of overtime up to 100 hours per month. This finding is in line with that in a prospective study of Japanese [16], showing a decreased risk of diabetes with increasing working hours (from <8 hours to ≥11 hours per day, which approximately correspond to overtime working from none to ≥60 hours per month, respectively). Another Japanese study observed no association in diabetes risk between employees working <9 hours and ≥9 hours per day (approximately monthly overtime work <20 hours and ≥20 hours, respectively) [15]. By contrast, another Japanese study found increased risk of diabetes among men who worked overtime >50 hours per month than those who worked less than 25 hours of overtime [13]. A study in the US [14] reported an increased risk of diabetes among those who worked 41 to 60 hours per week (approximately 4 to 80 hours of overtime per month); and no increase in the risk among those with overtime working ≥84 hours per month than those with no overtime work (working 21 to 40 hours per week). In addition, a recent meta-analysis of the cohort studies found that long working hours were not associated with the increased risk of diabetes [22]. Although there is no plausible explanation for the mixed results among studies, characteristics of subjects (white collar [16], local government employee [15], mostly blue collar [13], nurse [14], and mixture of white and blue collar in the present study) might have contributed to the inconsistent findings. In addition, the differences in the cutoff of overtime work hours may have resulted in the discordant results.

Reverse causality is a concern for cross-sectional studies, but it may not explain the present finding of a low diabetes prevalence associated with long overtime due to the following reasons. In Japan, occupational health system to prevent diseases related to long working hours has recently been introduced [18], including health guidance by occupational physician for workers with long working hours and restriction of working hours of employees with diseases [18]. Such preventive measures may have lead to the increased prevalence of diabetes among employees with short overtime. In the participating companies, however, occupational physicians recommend employers to shorten working hours only for patients in the advanced stage of disease including diabetes. In addition, we confirmed that an exclusion of subjects under medication for diabetes did not appreciably change the result. Alternatively, the present finding might be explained by risk factors for diabetes. Of risk factors we measured, short sleep duration and leisure time physical inactivity were associated with long working hours, and BMI was not. Although smoking rate was decreased with increasing working hours in the present study, adjustment for smoking did not seemingly change the results. Some other factors may contribute to the low diabetes prevalence associated with overtime. A recent Japanese study reported that daily total physical activity level, a protective factor for diabetes [23], was increased with increasing daily overtime working hours [16], suggesting some benefit of increased occupational physical activity on glucose metabolism.

In the present study, overtime working ≥100 hours per month was not associated with either increased or decreased odds of prevalent diabetes. To our knowledge, no previous study has examined the association with such long overtime. Nonetheless, our finding is in line with a study in the US [14], showing no increase in diabetes risk among those who worked ≥61 hours per week (≥84 hours of overtime per month; relative risk, 1.1). We have no clear explanation for the lack of increase in the prevalent diabetes among employees who not only worked extremely long hours but also had unfavorable lifestyles (short sleep hours and physical inactivity on leisure). As mentioned above, health guidance by a doctor for employees with long overtime working is required by law [18] and might have lead to the null finding; however, sensitivity analysis did not appreciably change the result. Our data did not support a view that extremely long overtime deteriorates glucose metabolism.

The strengths of our study include a large-scale data, and diagnosis of diabetes with a combination of fasting plasma glucose, HbA1c, and self-reported medication. Previous studies diagnosed diabetes by self-report [14], screening glycosuria followed by oral glucose tolerance test [13], or fasting glucose [15], [16]. Japan is among the countries with long working hours [8], and this allows us to examine the association with extremely long overtime work (100 hours per month or more). The cutoff for the longest overtime work category in the present study was much longer than those in previous studies, varying from 20 hours to 84 hours per month [13], [14], [15], [16]. Nevertheless, this study has several limitations. First, we cannot infer causal-relationship from results of a cross-sectional study. Second, because overtime work was self-reported by using different response options across companies, we chose overtime of <45 hours per month as the reference. In two companies where detailed data on working hour or overtime was available, however, an analysis using other definition of the reference (working 8 hours per day, which corresponds to no overtime, and 0 to 10 hours of overtime per month) showed similar result, at least up to 80 hours of overtime per month. Third, overtime in the past 1 to 3 months, depending on companies, was assessed only at one time point. Thus, the relationship between long-term exposure to overtime work and diabetes remains elusive. Fourth, a large number of subjects (23,573 out of 64,434) were excluded from the present analysis, and their majority (70%) were those aged less than 40 years, who are not required to receive blood test by law (except age 35 years). We cannot deny a possibility of bias associated with such selective inclusion. Fifth, healthy individuals, who have low risk of disease including diabetes, may tend to work long hours. If so, the association between overtime and prevalence of diabetes would be underestimated. Sixth, the present study was conducted among the large companies. Therefore, caution is needed to generalize the present finding to the small and middle-sized companies. Finally, even though we adjusted for potential confounders, unmeasured confounders including dietary factors, socioeconomic status, and work stress may have influenced the overtime work hours - diabetes association.

In conclusion, this cross-sectional analysis of large-scale data from Japanese workers showed a U-shaped association of overtime work with prevalent diabetes, with no increase in diabetes even among those who worked 100 hours or more of overtime per month, suggesting that overtime work may not be associated with impaired glucose metabolism. The present finding requires confirmation in longitudinal studies.
